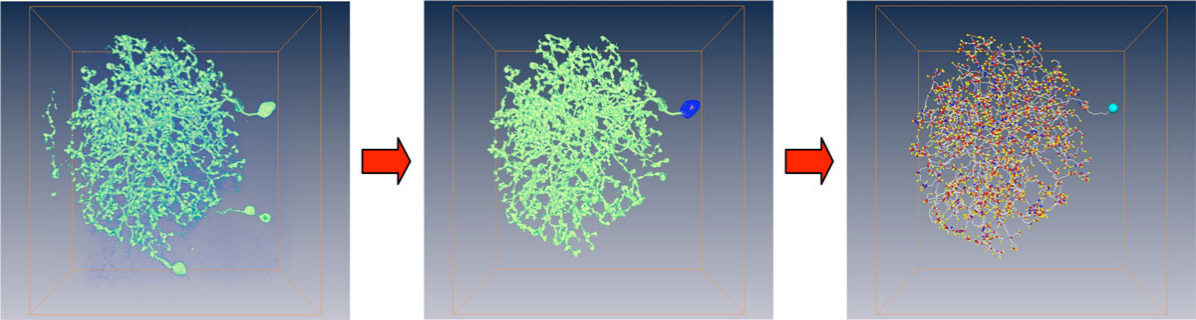# Large-scale segmentation and tracing for neurons in *Drosophila *brain by Fast Automatically Structural Tracing Algorithm (FASTA)

**DOI:** 10.1186/1471-2202-14-S1-P358

**Published:** 2013-07-08

**Authors:** Nan-Yow Chen, Meng-Fu Maxwell Shih, Chi-Tin Shih, Guan-Wei He, Ting-Yuan Wang, Li-An Chu, Wen-Wei Liao, Yu-Tai Ching, Ting-Kuo Lee, Ann-Shyn Chiang

**Affiliations:** 1 National Center for High-Performance Computing, Hsinchu 30076, Taiwan, R.O.C; 2 Department of Life Science, National Tsing Hua University, Hsinchu 30013, Taiwan, R.O.C; 3 Department of Physics, Tunghai University, Taichung 40704, Taiwan, R.O.C; 4 Department of Computer Science, National Chiao Tung University, Hsinchu 30010, Taiwan, R.O.C; 5 Institute of Plant and Microbial Biology, Academia Sinica, Taipei 11529, Taiwan, R.O.C; 6 Institute of Physics, Academia Sinica, Taipei 11529, Taiwan, R.O.C

## 

Recently, numerous three-dimensional neural images in *Drosophila *brains were taken from confocal laser scanning microscope [1]. However, how to obtain useful neuronal information from these messy raw data is very challenging. In order to conquer this problem, two critical issues need to be addressed: the first is to segment the image of single neuron from image data; the second is to trace the neuron fibers for quantitative analysis. Therefore, a robust segmentation process and an efficient tracing algorithm for single neuron are crucial and very desirable. At present there are methods and commercial software packages for these functions. But it requires a viewer to use his/her vision and judgment to segment and trace the neurons. Not only the task is very labor intensive but also the result is susceptible to errors and is usually lack of objectivity. Here we proposed an automatic procedure to segment and trace neural image data on a large scale. We first developed a new algorithm, Fast Automatically Structural Tracing Algorithm (FASTA), which encodes all image voxels on the idea of source field method and traces whole neuron via these codelets with some stopping criteria. Then, all image data were segmented into several single-neuron images with reasonable intensity threshold by using of FASTA iteratively. With this automatic procedure, single-neuron images and their annotations as well as quantified characteristics can be facilely and reliably retrieved as useful data for computational neuroscience.

**Figure 1:**